# Crystal Structure of Refolding Fusion Core of Lassa Virus GP2 and Design of Lassa Virus Fusion Inhibitors

**DOI:** 10.3389/fmicb.2019.01829

**Published:** 2019-08-13

**Authors:** Xuejiao Zhang, Cong Wang, Baohua Chen, Qian Wang, Wei Xu, Sheng Ye, Shibo Jiang, Yun Zhu, Rongguang Zhang

**Affiliations:** ^1^National Laboratory of Biomacromolecules, Institute of Biophysics, Chinese Academy of Sciences, Beijing, China; ^2^College of Life Sciences, University of Chinese Academy of Sciences, Beijing, China; ^3^Key Laboratory of Medical Molecular Virology of MOE/MOH/CAMS, School of Basic Medical Sciences and Shanghai Public Health Clinical Center, Fudan-Jinbo Joint Research Center, Fudan University, Shanghai, China; ^4^Interdisciplinary Innovation Institute of Medicine and Engineering, Beijing Advanced Innovation Center for Big Data-Based Precision Medicine, School of Biological Science and Medical Engineering, Beihang University, Beijing, China; ^5^Lindsley F. Kimball Research Institute, New York Blood Center, New York, NY, United States; ^6^National Center for Protein Science Shanghai, Shanghai Institute of Biochemistry and Cell Biology, Shanghai Institutes for Biological Sciences, Chinese Academy of Sciences, Shanghai, China

**Keywords:** Lassa virus, GP2, fusion core, crystal structure, viral entry

## Abstract

The envelope glycoproteins GP1 and GP2 of Lassa virus (LASV) bind to the host cell receptors to mediate viral infection. So far, no approved vaccines and specific treatment options against LASV exist. To develop specific fusion inhibitors against LASV, we solved the crystal structure of the post-fusion 6 helix bundle (6-HB) formed by two heptad repeat domains (HR1 and HR2) of GP2. This fusion core contains a parallel trimeric coiled-coil of three HR1 helices, around which three HR2 helices are entwined in an antiparallel manner. Various hydrophobic and charged interactions form between HR1 and HR2 domains to stabilize the overall conformation of GP2 fusion core. Based on the structure, we designed several peptides spanning the HR2 domain and tested their antiviral activities. We found that the longer HR2 peptides were effective in inhibiting LASV GPC protein-mediated cell–cell fusion under low pH condition. These results not only suggest that LASV infects the target cell mainly through endocytosis, including micropinocytosis, and membrane fusion at low pH, but also provide an important basis for rational design of LASV fusion inhibitors.

## Introduction

Lassa fever is an acute viral hemorrhagic illness occurring in West Africa, having posed a serious public health threat in many countries (Sogoba et al., 2012; Shaffer et al., 2014). Its case-fatality rate is 1% for overall infection and 15∼20% for severe cases among patients hospitalized. This mortality rate will increase sharply during epidemics or in pregnant women (Asogun et al., 2012). The etiologic agent of Lassa fever is Lassa virus (LASV), belonging to the arenavirus family. Arenavirus has more than 30 members divided into two groups: the New World viruses (or Tacaribe complex) and the Old World viruses (or LCM-Lassa complex). The New World family mainly contains Venezuelan hemorrhagic fever (VHF), Junín virus (JUNV), Machupo virus (MACV), and Bolivian hemorrhagic fever (BHF), while the Old World viruses includes, for example Lujo virus (LUJV), Lymphocytic choriomeningitis virus (LCMV), Morogoro virus (MORV), and LASV. These arenaviruses have both geographical and genetic differences.

Lassa virus is an enveloped, single-stranded RNA virus. The two RNA segments in its genome encode four viral proteins, including zinc-binding protein (Z), RNA polymerase (L), nucleoprotein (NP), and the surface glycoprotein precursor (GP, or spike protein). GP is cleaved into envelope glycoproteins GP1 and GP2 (Cao et al., 1998). GP1 that is responsible for receptor binding (including α-dystroglycan, heparin sulfate, DC-SIGN, etc.) and GP2 that mediates membrane fusion interact with each other to form a stable trimer complex on the LASV viral envelope (Li et al., 2016; Hastie et al., 2017). Upon receptor binding, LASV enters the target cell via clathrin- and dynamin-independent endocytosis with subsequent transport to late endosomal compartments, where fusion occurs at low pH (Vela et al., 2007; Rojek et al., 2008). A recent study has also found that after GP1 binds to cell receptor α-dystroglycan, LASV enters into the target cell through the unusual micropinocytosis pathway and membrane fusion under low pH condition (Oppliger et al., 2016).

So far, no approved vaccines and specific treatment modalities against LASV are available. Given that the first peptide-based antiviral drug enfuvirtide (T20) inhibits human immunodeficiency virus (HIV) fusion with and entry into the target cell by targeting the heptad repeat domain in the viral envelope glycoprotein gp41 (Qi et al., 2017; Su et al., 2017), the heptad repeat domain in GP2 of LASV may also serve as a target for the design of LASV fusion inhibitors. We have previously demonstrated that the N-terminal heptad repeat 1 (HR1) domain binds to C-terminal heptad repeat 2 (HR2) domain to form a stable six-helix bundle (6-HB) to mediate viral entry; therefore, peptides derived from HR2 should specifically bind to the homotrimeric HR1, interfering with the formation of 6-HB and, hence, blocking viral entry (Zhu et al., 2015, 2016, Xia et al., 2019). Therefore, it is essential to determine the 6-HB core structure and characterize the interaction sites in the HR1 and HR2 domains, in order to design the HR2-derived peptides against LASV infection. Here, we solved the crystal structure of the post-fusion 6-HB formed by LASV HR1 and HR2 domains. Based on the structure, we designed several peptides spanning the HR2 domain and tested their antiviral activities. Concurrent with the preparation of the present manuscript, another group reported a post-fusion structure of LASV using the insect baculovirus expression system (Shulman et al., 2019), which allows us to compare the structural differences of 6-HB cores formed by the HR1 and HR2 domains in their truncated version of LASV GP2 spanning residues 306–421 with those in our LASV HR1-T-loop-HR2 construct. These comparisons provide more comprehensive knowledge for better understanding the entry mechanism of LASV and designing of peptide-based LASV fusion inhibitors.

## Materials and Methods

### Cells, Peptides, and Plasmids

The 293T cell line was obtained from ATCC (Manassas, VA, United States), and the Huh-7 cell line was from the Cell Bank of the Chinese Academy of Sciences (Shanghai, China). These two cell lines were propagated in Dulbecco’s Modified Eagle’s Medium (DMEM) supplemented with 10% fetal bovine serum (FBS). Peptides (LASV/HR2-1, LASV/HR2-2, LASV/HR2-3, LASV/HR2-4, and LASV/HR2-5) were synthesized by solid-phase peptide synthesis at SYN Inc. (Shanghai, China). Recombinant plasmids encoding the LASV GPC protein were synthesized by SYN Inc.

### Protein Expression and Renaturation

The gene coding for HR1-T-loop-HR2 construct of LASV GP2 (residues 306–432, with a C310S mutation and Δ_378_GK_379_ truncation) was amplified by PCR and cloned into vector pET-28a with an artificially introduced PreScission Protease cleavage site at its N-terminus for stable expression. The fusion protein was overexpressed in *Escherichia coli* BL21. Cells were grown to OD600 ≈ 0.6 in lysogeny broth (LB) media supplemented with 1 μg/mL kanamycin at 37°C and were induced by 1 mM IPTG for 16 h at 37°C for expression. Cells were harvested by centrifugation at 4500 × *g* for 15 min at 4°C and were lysed by high pressure homogenizer twice after resuspension in buffer containing 25 mM Tris–HCl, pH 8.0, and 200 mM NaCl. The inclusion body was harvested by centrifugation at 18,000 x*g* for 30 min and resuspended in buffer containing 50 mM Tris–HCl, 200 mM NaCl, pH 8.0, 8 M Urea, and 50 mM DTT. Then the trimeric LASV 6-HB protein was refolded using limit dilution method. Briefly, the denatured protein was diluted at 1:100 volume ratio into renaturation buffer (25 mM Tris–HCl, 200 mM NaCl, pH 8.0, 100 mM Arginine, 1 mM GSH and 0.1 mM GSSG) very slowly (0.02 mL/min), and stored for 1 week at 4°C. Refolded protein was isolated by Ni-affinity chromatography and purified by anion exchange chromatography (HiTrap Q Fast Flow 5 mL, GE Healthcare), as well as gel filtration chromatography (Superdex 200 10/300 GL, GE Healthcare), and then concentrated to 15 mg/mL for crystallization or storage at −80°C for further use.

### Crystallization

Crystals were obtained at 16°C for 7 days using the hanging drop vapor diffusion method by mixing equal volume of protein solution [LASV-6-HB: 6 mg/mL] and reservoir solution, [0.17 M Ammonium Acetate, 0.085 M Sodium Citrate: HCl, pH 5.6, 25.5% (w/v) PEG 4000, and 15% (v/v) Glycerol]. Then crystals were flash-frozen after immersing in paraffin oil for about 10 s, followed by transfer to liquid nitrogen for further data collection.

### Data Collection, Structure Determination, and Refinement

The datasets were collected at beamline BL-18U1, Shanghai Synchrotron Radiation Facility, at a wavelength of 0.97930 Å. The crystals were kept at 100 K during X-ray diffraction data collection. Data were indexed and scaled with HKL2000 (Otwinowski and Minor, 1997). Phases were solved by the molecular replacement method using PHENIX.phaser (Mccoy, 2007). All refinement procedures were carried out with PHENIX.refine (Zwart et al., 2008) and COOT (Emsley and Cowtan, 2004). Table 1 shows the detailed statistics of data collection and refinement.

**TABLE 1:** Data collection and refinement statistics.

	**LASV 6-HB PDB ID: 6JGY**
**Data collection**	
Space group	H32
**Cell dimensions**	
a, b, c (Å)	58.7, 58.7, 268.6
α, β, γ (°)	90, 90, 120
Wavelength (Å)	0.97930
Resolution (Å)	3.39–38.04 (3.39–3.48)^†^
*R*_merge_	0.118 (0.080)
Mean I/σ(I)	19.2 (3.4)
Completeness (%)	98.1 (100.0)
Redundancy	6.3 (6.4)
**Refinement**	
Resolution (Å)	3.39–38.04
No. of reflections	2839
Reflections in test set	135
*R*_work_/R_free_	0.233/0.274
**No. of atoms**	
Protein	873
Water	0
**R.m.s. deviations**	
Bond lengths (Å)	0.010
Bond angles (°)	1.214
Average *B*-factor (Å^2^)	103.01

*^†^Values in parentheses are for the highest resolution shell.*

### Accession Numbers

Coordinates and structure factors have been deposited in the Protein Data Bank with accession number 6JGY for crystal structure of fusion core of LASV GP2 protein.

### Biolayer Interferometry

Biolayer interferometry (BLI) is a common technique for detecting interactions between substances. The working principle of this method is that the molecules are immobilized on the surface of the sensor to form a biolayer, thereby causing interference against light waves passing through the sensor, which was then detected in the form of phase displacement, so that any change in the number of molecules on the surface of the sensor can be detected. Based on this principle, the mobile phase cannot have a non-specific combination with the sensor. The sensor type selected in this experiment was Super Streptavidin (SSA) Biosensor, and all the tests were performed using Octet RED96.

The biotinylation of protein for immobilization onto SSA was carried out according to the following procedure. The activated biotin reagent was prepared in DMSO at a concentration of 5 mg/mL. The peptides were mixed with biotin reagent, and the molar ratio of peptide to biotin reagent was 2:1. The reactions were incubated at room temperature for 1 h. Then free biotin was removed by dialysis. Biotinylation of protein for immobilization onto SSA was at a concentration of 80 μg/mL. The loading time was 300 s. The concentration of mobile phase in the initial test was 50 μM. The concentration gradient in the further test was 30, 15, 7.5, and 3.75 μM. The association time was 300 s, and the dissociation time was 600 s.

### CD Spectroscopy

The secondary structure of peptides LASV/HR2-1 (residues 385–432) and LASV/HR1 (residues 296–345), as well as their mixture, were determined by CD spectroscopy. Briefly, the peptides were diluted in phosphate-buffered saline (PBS) (pH 7.2). After incubating at 37°C for 30 min, CD spectra were acquired on a Jasco spectropolarimeter (model J-815; Jasco, Inc., Easton, MD, United States) from 195 to 260 nm at room temperature, using the optical path length of 0.1 cm and bandwidth of 1 nm. The baseline was determined using PBS. The α-helical content was calculated from the CD signal by dividing the mean residue ellipticity [θ] at 222 nm by the value expected for 100% helical formation (−33,000 degrees cm^2^ dmol^–1^).

### Production of Pseudoviruses

Lassa virus pseudoviruses were constructed as described previously (Li et al., 2017; Wang et al., 2017). Briefly, 293T cells were seeded in a 10-cm tissue culture dish. When 293T cells grown in a 10-cm dish reached 80% confluence, cells were cotransfected with plasmids pcDNA3.1-LASV-GPC (encoding GPC protein of LASV) and pNL4-3.luc.RE (encoding Env-defective, luciferase-expressing HIV-1 capsid protein) at a ratio of 1:1 using VigoFect (Vigorous Biotechnology, Beijing, China). The supernatant was replaced with fresh DMEM at 8–10 h post-transfection and harvested after incubation for an additional 72 h. Cell debris was removed by centrifuging at 3000 rpm for 10 min, followed by filtration through a 0.45 μm filter.

### Inhibition of LASV Pseudovirus Entry Into the Target Cells

A LASV pseudovirus inhibition assay was performed in a manner similar to other envelope virus assays (Lu et al., 2014; Channappanavar et al., 2015; Xia et al., 2018). Briefly, Huh-7 cells were placed (10^4^ cells/well) into a 96-well plate and incubated overnight at 37°C. LASV pseudovirus was incubated with serially diluted peptides for 30 min at 37°C, followed by the addition of Huh-7 cells. The cells were incubated with or without pseudovirus as virus control and cell control, respectively. At 12 h post-infection, the culture was replaced with fresh medium, followed by an additional incubation for 72 h. Cells were lysed, and cell lysates were transferred to a 96-well Costar flat-bottom luminometer plate (Corning Costar, New York, NY, United States), followed by the addition of luciferase substrate (Promega) to measure luminescence using an Infinite M200 PRO (Tecan, GröDig, Austria).

### Inhibition of LASV GPC Protein-Mediated Cell–Cell Fusion

Lassa virus GPC protein-mediated cell–cell fusion was performed as previously described (Cosset et al., 2009). Briefly, plasmid pAAV-IRES-LASV-EGFP encoding the LASV GPC protein was transfected into 293T cells (293T/LASV/EGFP) using the transfection reagent VigoFect (Vigorous). When GFP was obviously expressed on most 293T cells, 293T/LASV/EGFP cells were digested and mixed with Huh-7 cells at ratio of 1:1. The mixture was incubated at 2 × 10^4^ cells/well in wells of a 96-well plate for 12 h. The peptides were serially diluted with low pH (pH5) DMEM and then added to the mixture of 293T/LASV/EGFP cells and Huh-7 cells. After 20 min of exposure in the low pH medium for triggering the fusion between the 293T/LASV/EGFP cells and Huh-7 cells, the cells were restored to neutral medium and cultured for 1 to 2 h at 37°C. The 293T/LASV/EGFP cells fused or unfused with Huh-7 cells were fixed with 4% PFA and counted under an inverted fluorescence microscope (Nikon, Tokyo, Japan). The fused cells showed much larger size and weaker fluorescence intensity than the unfused cells because of the diffusion of EGFP from one cell to more cells.

## Results

### Overall Structure of LASV Fusion Core

**FIGURE 1:**
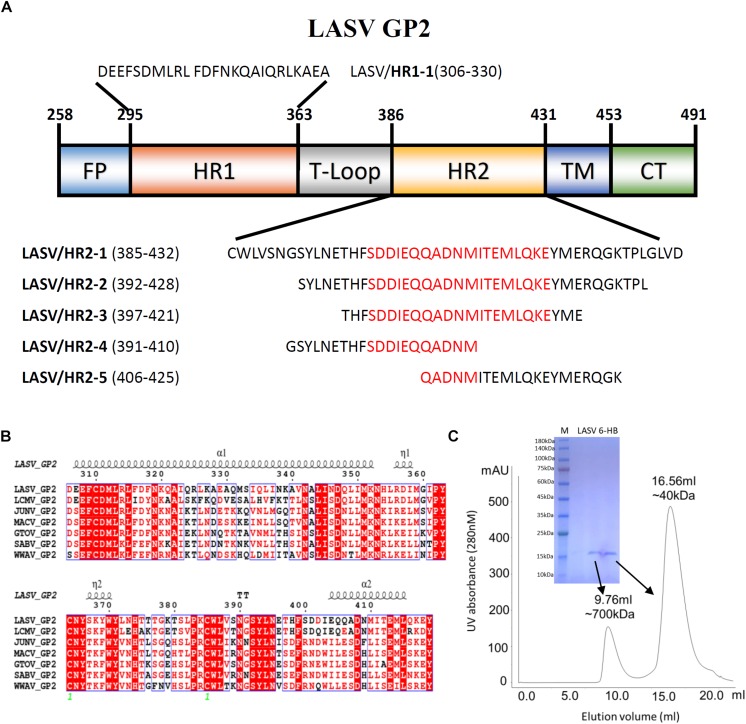
The amino acid sequence and purification of LASV fusion core. **(A)** The division of different domains of LASV GP2 protein, including fusion peptide (FP), first heptad repeat domain (HR1), linker of T-loop domain, second heptad repeat domain (HR2), transmembrane domain (TM) and C-terminal intracellular domain (CT). The designed peptides are also listed. **(B)** Sequence alignment of GP2 from representative arenaviruses, like LASV (*Lassa virus*, Taxon ID: 11622), LCMV (*Lymphocytic choriomeningitis virus*, Taxon ID: 11624), JUNV (*Junin mammarenavirus*, Taxon ID: 2169991), MACV (*Machupo virus*, Taxon ID: 11628), GTOV (*Guanarito mammarenavirus*, Taxon ID: 45219), SABV (*Sabia mammarenavirus*, Taxon ID: 2169992), and WWAV (*Whitewater Arroyo mammarenavirus*, Taxon ID: 46919). **(C)** The size exclusion chromatography and SDS–PAGE result of refolded LASV GP2 6-HB proteins.

The GP2 protein contains a N-terminal fusion peptide (FP) (residues 258-295), a N-terminal HR1 domain (residues 295-363), a linker T-loop domain (residues 363-386), a C-terminal HR2 domain (residues 386-431), a transmembrane domain (residues 431-453), and an intracellular domain (residues 454-491) (Figure 1A). By multiple sequence alignment with other representative arenaviruses, like LCMV, JUNV, and MACV, LASV showed a highly conserved T-loop domain and a variable HR2 domain (Figure 1B). Two ends of the HR1 domain are conserved, but its middle region is relatively unique, which may be helpful to adapt the variable HR2 domain of LASV GP2.

To reveal the interaction between HR1 and HR2 domains of LASV, stable 6-HB covering HR1, T-loop and HR2 domains (residues 306–432), was constructed for crystallographic study. The denaturation and renaturation were used to acquire stable 6-HB from inclusion body to mimic its post-fusion state. During refolding process, the wild type LASV 6-HB protein mainly formed precipitations or high polymers. After the single mutation of C310S was introduced, the trimeric LASV 6-HB protein was obtained (Figure 1C). However, the large-scale of crystal screening for this protein could not yield high-quality crystals. Then we continued to try different truncations and deletions in 6-HB, and finally found that a deletion of Gly378 and Lys379 in the T-loop region could yield protein crystal with enough quality for structure determination.

**FIGURE 2:**
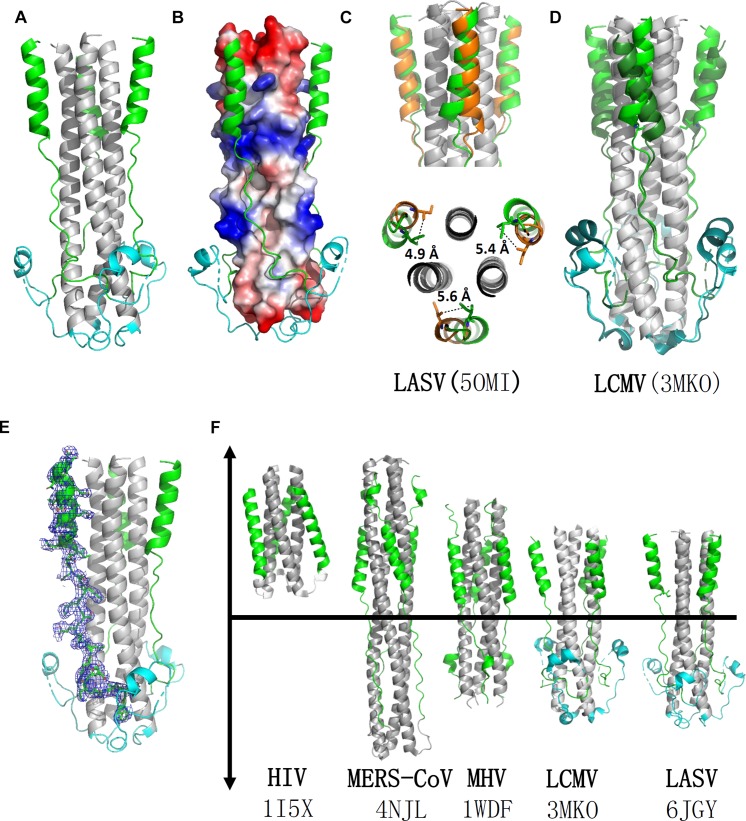
Crystal structure of LASV fusion core. **(A)** The overall structure of HR1-T-loop-HR2 domains of LASV is shown in cartoon representation. HR1 domain is colored in gray, T-loop is in cyan and HR2 is in green. **(B)** The HR1 trimer core is shown as electrostatic potential surface. **(C)** Comparing the structure with another reported LASV 6-HB structure (PDB: 5OMI), the HR2 domain of which is colored in orange. **(D)** Comparing the structure with fusion core structure of LCMV (PDB: 3MKO), the T-loop domain and HR2 domain of which are colored in dark green. **(E)** The electron density map of HR2 domain are shown as blue mesh. **(F)** Structural comparison of different fusion cores, including HIV (PDB: 1I5X), MERS-CoV (4NJL), MHV (PDB: 1WDF), LCMV (PDB: 3MKO), and LASV (PDB: 6JGY). Their HR1 domains are colored in gray, HR2 in green, and T-loop in cyan.

The overall structure of the HR1-T-loop-HR2 domain showed a canonical 6-HB structure (Figure 2A). Taking a rod-like shape with a length of ∼75 Å and a diameter of ∼40 Å, the LASV fusion core contains a parallel trimeric coiled-coil of three HR1 helices (gray in Figure 2A), around which three HR2 helices are entwined (green in Figure 2A) in an antiparallel manner. Between these two domains, the T-loop forms a 3_10_ helix linker hovering outside (cyan in Figure 2A).

### Comparison of the Fusion Core Structure of LASV With Those of LCMV, HIV, Middle East Respiratory Syndrome Coronavirus (MERS-CoV), and Mouse Hepatitis Virus (MHV)

According to the structure shown in electrostatic potential surface (Figure 2B), the HR2 region of LASV can be divided into two parts: a linear N-terminal region and a helical C-terminal region. The linear N-terminal region binds to two adjacent HR1 helices through hydrophobic interactions, consistent with other fusion core structures, as previously determined (Lu et al., 2014). Some obvious charged interactions take place between the helical C-terminal region of HR2 and HR1 trimer core, and these will be discussed later. Another group deposited a post-fusion structure of LASV in PDB (entry code 5OMI), using a baculovirus expression system, instead of our renaturation method. Compared with this structure, the HR2 helical region in our structure exhibited a significant shift of 4.9∼5.6 Å toward the hydrophobic grooves of two adjacent HR1 helices, showing much stronger hydrophobic interaction (Figures 2C,E).

Lymphocytic choriomeningitis virus and LASV both belong to the Old World family and have high sequence homology (Figure 1B). Comparing the fusion core structure of LCMV (PDB entry 3MKO) with that of LASV, we found that their HR1 domains overlap with each other very well, but their T-loop and HR2 helical regions have obvious differences (Figure 2D). The T-loop region of LCMV fusion core moves closer to the HR2 region, and its HR2 helical region slopes away from HR2 of LASV. When further comparing the 6-HB structures of LCMV and LASV with those of other viruses, we found several unique features for arenavirus GP2 protein (Figure 2F). The HR1 and HR2 regions of HIV form a short regular α-helix structure, while coronaviruses, like MERS-CoV and MHV, have a short helical HR2 region, but longer helical HR1 region. For arenavirus of LASV or LCMV, a special T-loop region is situated between HR1 and HR2 domains, packing along the linear HR2 region.

### Interactions in LASV 6-HB Fusion Core

**FIGURE 3:**
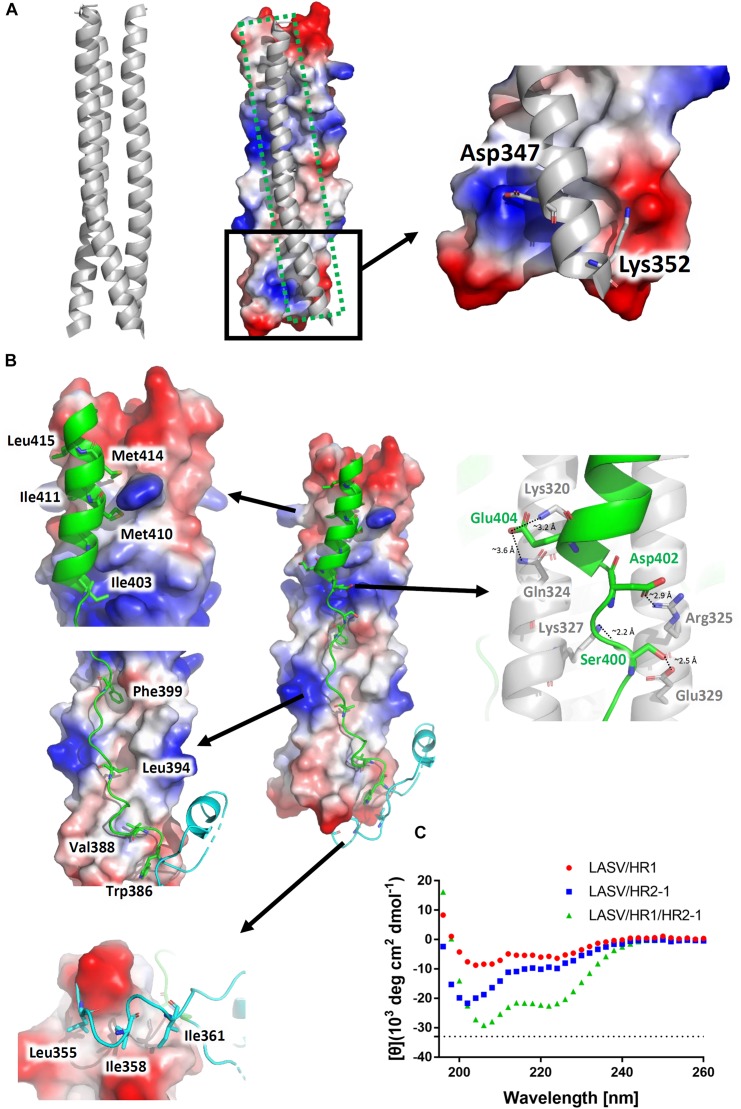
Interactions within the LASV 6-HB fusion core. **(A)** Three HR1 helices are tightly packed together in the fusion core with a buried interface of 1584 Å^2^ for each HR1. Hydrophilic interactions are also at the end of the trimers. **(B)** Interactions for T-Loop or HR2 domain against HR1 trimer core. The buried interface for each HR2 domain with HR1 core is 2143 Å^2^. Important hydrophobic and hydrophilic residues are indicated. **(C)** The circular dichroism spectroscopy of HR peptides.

The three HR1 helices of LASV are closely packed against each other by hydrophobic force in a parallel manner. The buried area for each HR1 domain reaches 1584 Å^2^, indicating a much stronger interaction (Figure 3A). At the C-terminal of HR1, it is interesting that two hydrophilic interactions occur among the HR1 trimers. The Asp347 and Lys352 of one HR1 domain bind to the residues with opposite charges in other two HR1 domains. As shown in Figure 1B, these two residues are highly conserved among different arenaviruses, suggesting that this additional charged interaction may play an important role in enhancing the stability at the end of HR1 trimer.

The T-loop region and HR2 domain are well packed against the hydrophobic grooves of a central three-helical coiled coil with an interface of 2143 Å^2^ (Figure 3B). Leu355, Ile358, and Ile361 of T-loop bind to the C-terminal end of HR1 trimer. In the linear HR2 domain, Trp386, Val388, Leu394, and Phe399 are deeply buried in the HR1 hydrophobic groove. Even in the helical HR2 domain, many hydrophobic residues with long side chains are involved in binding to HR1, including Ile403, Met410, Ile411, Met414, and Leu415 (Figure 3B). Moreover, at the junction region between linear and helical parts of HR2, several strong hydrogen bonds and salt bridges are noted. For hydrogen bonds, the hydroxyl group of Ser400 in HR2 interacts with Glu329 in one HR1 helix (∼2.5 Å distance), while its carbonyl oxygen interacts with Lys327 in another HR1 helix (∼2.2 Å distance) (Figure 3B). For salt bridges, Asp402 interacts with Arg325 (∼2.9 Å distance), and Glu404 interacts with Lys320 and Gln324 (∼3.2 Å and 3.6 Å distances, respectively). These relatively concentrated hydrophilic interactions constitute an anchoring point in the middle of HR2 domain, stabilizing the linear and helical region of HR2 and also the whole 6-HB conformation.

To confirm the secondary structure before and after the formation of fusion core, the α-helical ratios of HR1 peptide, HR2 peptide, and their mixture were analyzed by circular dichroism. In solution, the single HR1 and HR2 peptides showed relatively low α-helical ratio of 17 and 28%, respectively. However, when they were mixed together to form 6-HB, the α-helical ratio largely increased to 68% (Figure 3C), which is consistent with the crystal structure. It suggests that the two peptides undergo a significantly conformational change when they interact with each other to form 6-HB fusion core.

### Biophysical Characterization of HR2-Derived Peptides

**FIGURE 4:**
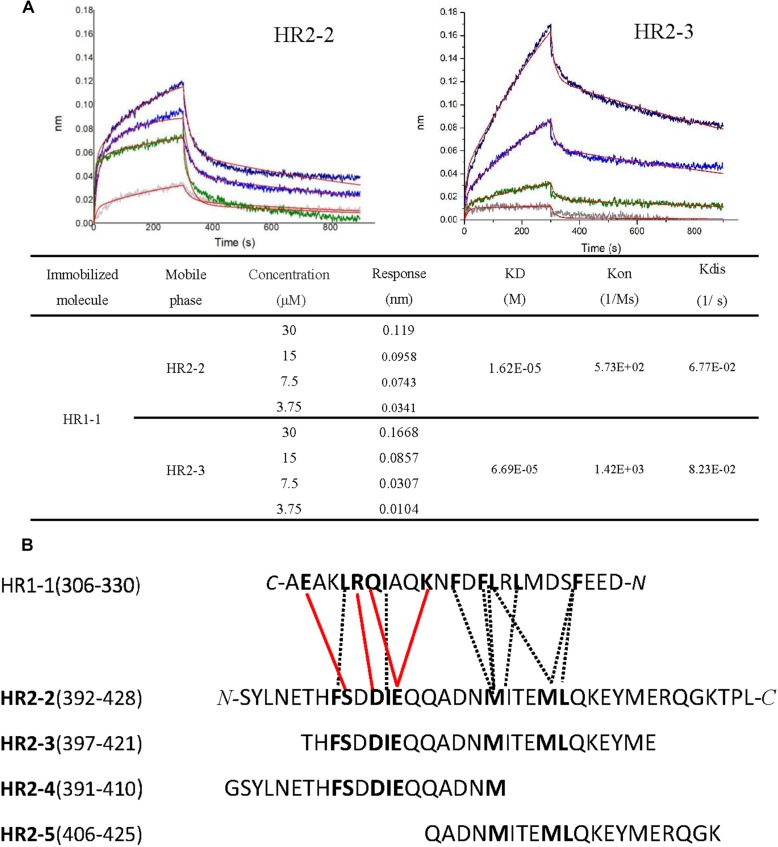
Binding affinity between HR1 and HR2 peptides was measured by BLI. **(A)** Binding curve of HR2-2 or HR2-3 peptide in concentration gradient (30 μM, 15 μM, 7.5 μM, and 3.75 μM) against HR1-1 peptide. The red curves represent the fitting line. The KD values are also shown. **(B)** The interaction residues between HR1- and HR2-derived peptides. The hydrophobic interactions are shown in black dashed lines, while the hydrophilic interactions are shown in red lines.

To elucidate the interactions between HR1 and HR2 regions of LASV, as observed in the crystal structure, the HR1-derived peptide (HR1-1) and HR2-derived peptides (HR2-1∼HR2-5) were synthesized to measure their binding affinities using BLI. HR2-1 peptide showed a significant non-specific adsorption on the sensor, making it impossible to measure affinity data. All other peptides showed weak non-specific binding to the sensor at the concentration of 60 μM. Then, HR1-1 was used as the immobilized molecule to detect the affinity between HR1-1 and HR2-2 ∼ HR2-5. The results showed that HR2-2 and HR2-3 peptides could strongly bind to HR1-1 peptides in a dose-dependent manner (Figure 4A), while HR2-4 and HR2-5 could not.

Both hydrophobic and hydrophilic interactions between HR2-4 and HR1-1 peptide are seen in the crystal structure (Figure 4B). The major hydrophobic interactions in HR2-4 peptide (391-410) include Phe399, Ile403, and Met410, which were buried in the hydrophobic groove of HR1-1 trimeric core. Several hydrogen bonds were also observed between HR2-4 and HR1-1. The side chain oxygen of Ser400 binds to Glu329 in one HR1 helix, while its main chain oxygen binds to Lys327 in another HR1 helix (Figure 3B). Then Asp402 binds to Arg325, and Glu404 interacts with Lys320 and Gln324. However, despite these potential interactions, BLI results showed weak interaction between HR2-4 and HR1-1 peptides. It is possible that a short peptide like HR2-4 may not form the right conformation to bind to its target; otherwise, the missing hydrophobic interactions mediated by Met414 and Leu415 might reduce the binding affinity.

Almost no hydrophilic interaction occurs between the HR2-5 peptide and HR1-1 peptide in the structure. Their hydrophobic interactions are mainly provided by Met410, Ile411, Met414, and Leu415 (Figure 3B). Based on BLI testing, no strong interactions were found between HR2-5 and HR1-1 peptides. Therefore, the missing hydrogen bonds and hydrophobic residues largely reduce HR2-5′s interactions with viral HR1 core.

In the BLI test, both HR2-2 and HR2-3 peptides exhibited interactions with HR1-1 peptide. The structure also showed many strong hydrophobic and hydrophilic interactions between HR2-2 and HR2-3 peptides, both of which have identical residues providing hydrophobic interactions with HR1-1, including Phe399, Ile403, Met410, Ile411, Met414, and Leu415 (Figure 3B). They also share the same amino acids involved in hydrophilic interactions, including Ser400, Asp402, and Glu404. Compared with HR2-4 and HR2-5, longer peptides like HR2-2 and HR2-3 exhibited the optimal conformation folding and had sufficient key residues for interaction with the HR1-1 trimer.

### The Longer HR2-Peptides Could Not Block LASV Pseudovirus Entry Into the Target Cell, but Were Effective in Inhibiting LASV GPC Protein-Mediated Cell–Cell Fusion

**FIGURE 5:**
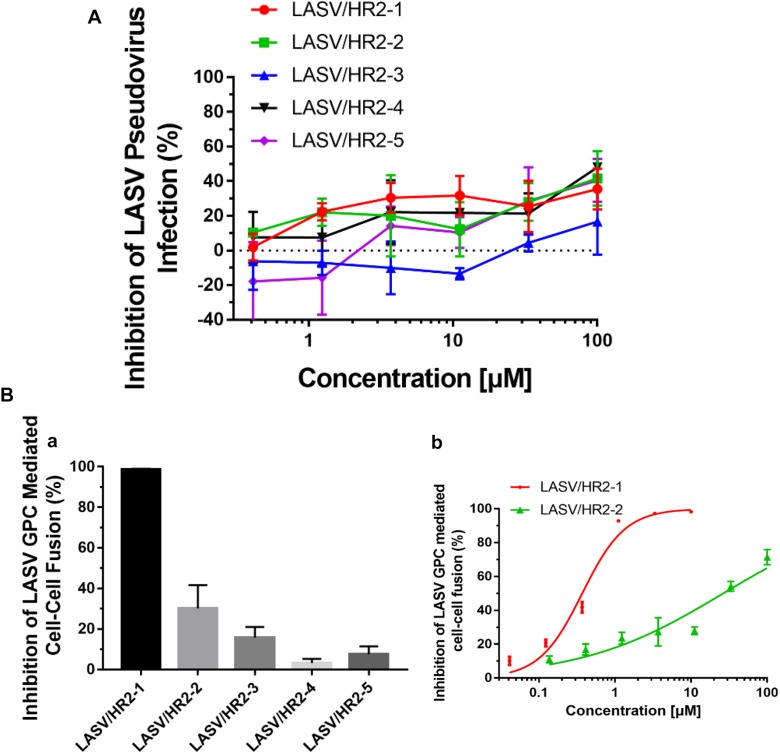
The inhibitory activity of LASV HR2-derived peptides on LASV pseudovirus into the target cell and LASV G protein-mediated cell–cell fusion. **(A)** Inhibition of HR2-derived peptides on the entry of LASV pseudovirus into the host cell. **(B)** Inhibition of five HR2 peptides at 20 μM **(a)** or different concentrations **(b)** against LASV GPC protein-mediated cell–cell fusion.

Because of the strict restriction on the use of highly pathogenic viruses in our BSL-3 facilities, we were not able to get live LASV for testing the anti-LASV activity of the HR2-peptides. We thus tested the potential inhibitory activity of these peptides against LASV pseudovirus entry into the target cells. Unexpectedly, none of the HR2-derived peptides exhibited significant inhibitory activity on the entry of the LASV pseudovirus into the target cell at the concentration as high 100 μM (Figure 5A). These results suggest that the HR2-peptides may not interact with the GP1 protein, which mediates the attachment of LASV to the target cell, the first step of viral entry, and that LASV may not get into the cell through the cytoplasm membrane fusion under neutral pH condition, the second step of entry of some class I enveloped viruses, such as HIV and MERS-CoV (Qi et al., 2017; Su et al., 2017; Xia et al., 2019).

Next, we assessed the potential inhibitory activities of these HR2-derived peptides against LASV GPC protein-mediated cell–cell fusion under low pH condition. At 20 μM, peptide HR2-1 could completely inhibit the cell–cell fusion, while peptides HR2-2 and HR2-3 could inhibit about 30–40% and 20% cell–cell fusion, respectively, and the peptides HR2-4 and HR2-5 showed no inhibitory activity (Figure 5Ba). Further analysis indicated that the peptides HR2-1 and HR2-2 inhibited cell–cell fusion in a dose-dependent manner with the IC_50_ (the half maximal inhibitory concentration) values of 0.37 and 26.45 μM, respectively (Figure 5Bb), while other HR2-derived peptides had no detectable inhibitory activity. These results suggest that the longer HR2 peptides could inhibit cell–cell fusion because of their higher affinity to bind with the HR1 groove (Figure 3B) and that LASV may enters into the target cell through micropinocytosis and membrane fusion under low pH condition.

## Discussion

We solved the crystal structure of the post-fusion 6-HB formed by HR1 and HR2 domains of LASV GP2 protein and then designed several HR2-derived peptides to study their binding affinities against HR1 peptide and inhibitory activities for viral entry. We found that the longer HR2 peptides, HR2-2 (37-mer: 392-428), and HR2-3 (25-mer: 397-421), had higher binding affinity with HR1 peptide than the shorter HR2 peptides, HR2-4 (20-mer: 391-410), and HR2-5 (20-mer: 406-425), possibly owing to their rich hydrophobic and hydrophilic interactions (Figure 4). Moreover, the longer peptides of HR2-1 (48-mer: 385–432) and HR2-2 exhibited more obvious inhibitory activities against LASV G protein-mediated cell–cell fusion. These results suggest that the longer HR2-derived peptides, which have stronger affinity against the HR1 domain, also have more potent inhibitory activity. On the contrary, the shorter HR2 peptides, HR2-4 and HR2-5, do not interact with HR1 peptide, thus showing no cell–cell fusion inhibitory activity. In the cell–cell fusion process, the GP1 protein of LASV binds to its target receptor to expose the GP2 subunit. Then, the HR2 domain binds to the HR1 domain to form the 6-HB fusion core to mediate the formation of membrane fusion pores. At this time, the HR1 domain becomes a very exposed target for binding and blocking by an HR2-derived peptide. Therefore, the binding affinity of HR2 peptides is positively related to the inhibitory activity against LASV GPC protein-mediated cell–cell fusion under low pH condition.

**FIGURE 6:**
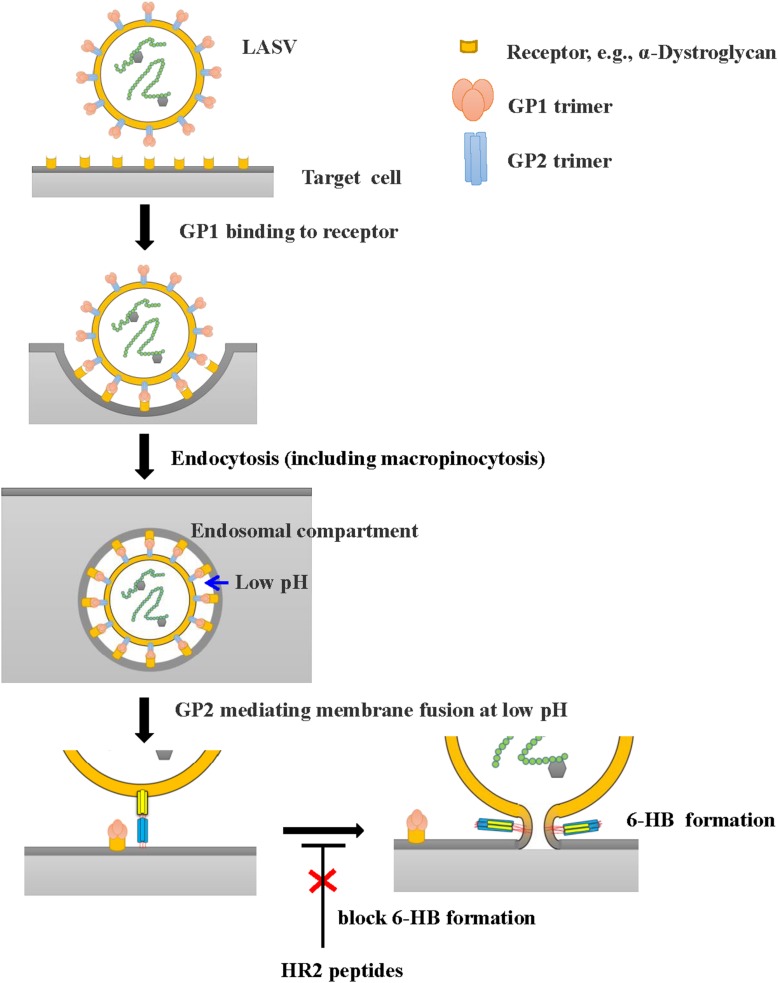
Proposed infection model of LASV. LASV can enter the target cell via endocytosis, including macropinocytosis, and membrane fusion at low pH. HR2-peptides or their analogs, if they can enter the endosomal compartment inside the host cell, can interact with the HR1 domain in viral GP2 and block the 6-HB formation, thereby inhibiting membrane fusion at low pH. Their fusion inhibitory activity can be assessed using the LASV GPC protein-mediated cell–cell fusion assay under low pH condition (Figure 5B).

However, all these HR2-derived peptides showed weak or no inhibitory activity against LASV pseudovirus entry into the target cell. It confirms that the HR2-peptides do not interact with the GP1 protein to block its interaction with the receptor on the target cell, the first step of viral entry. These findings also suggest that LASV enters the target cell using the fusion pathway different from that utilized by some other class I enveloped viruses, such as HIV and MERS-CoV (Qi et al., 2017; Su et al., 2017; Xia et al., 2019), which get into the host cells via cytoplasm membrane fusion under neutral pH condition. Several groups have shown that LASV enters the host cell via clathrin- and dynamin-independent endocytosis and membrane fusion occurs at low pH (Vela et al., 2007; Rojek et al., 2008). Oppliger and coworkers (Oppliger et al., 2016) have recently reported that LASV enters the target cell through macropinocytosis. Since our results are consistent with those in the reports above, we proposed an infection model of LASV (Figure 6). Specifically, LASV enters the target cells via endocytosis, including macropinocytosis. In the membrane fusion process as revealed by the cell–cell fusion assay, the GP1 trimer of LASV binds to its receptor(s), e.g., α-Dystroglycan, to trigger the conformational change of GP2. Then HR1 is exposed to bind with HR2 to form 6-HB, resulting in the fusion between viral envelope and endosomal membrane. Under these conditions, HR2-derived peptides could bind to the HR1 target to block viral infection. However, during the entry process of live and pseudotyped LASV, the HR1 domain of viral GP2 can only be exposed in the endosomal compartment and interacts with the HR2 domain of viral GP2 to mediate membrane fusion under low pH condition, making it impossible for HR2 peptides to enter the endosomal compartment to block membrane fusion there (Figure 6). Therefore, HR2 peptides must be modified, for example, by adding TAT cell penetration sequence (Miller et al., 2011) or hydrocarbon stapling motif (Wang et al., 2018), so that they can enter into the endosomal compartment inside the cell to interact with HR1 domain of the viral GP1 domain and inhibit the membrane fusion at low pH there.

In the BLI test, HR2-2 and HR2-3 peptides exhibited different binding affinity with HR1-1 peptide, even though they shared the same hydrophilic and hydrophobic residues to interact with NHR fusion core in the structure (Figure 4). HR2-2 peptide has several additional residues in both N-terminus (SYLNE) and C-terminus (RQGKTPL) compared with the HR2-3 peptide. Although these residues are not involved in the NHR-CHR interactions in the crystal structure, they may help stabilize the overall structure of the entire HR2 peptide. Thus, compared with the HR2-3 peptide, the more stable HR2-2 peptide has higher affinity in binding HR1-1 peptide (Figure 4), as well as higher inhibitory activity in cell–cell fusion (Figure 5B). Therefore, the HR2-2 peptide will be modified for further development as an anti-LASV drug candidate.

## Data Availability

The datasets generated for this study can be found in the Protein Data Bank with accession number 6JGY for crystal structure of fusion core of LASV GP2 protein.

## Author Contributions

YZ, SJ, and SY designed the experiments. YZ, SJ, and RZ wrote the manuscript. XZ and BC performed the protein purification and crystallization. CW, QW, and WX participated in the viral experiments.

## Conflict of Interest Statement

The authors declare that the research was conducted in the absence of any commercial or financial relationships that could be construed as a potential conflict of interest.
